# Acute Effect of Pistachio Intake on Postprandial Glycemic and Gut Hormone Responses in Women With Gestational Diabetes or Gestational Impaired Glucose Tolerance: A Randomized, Controlled, Crossover Study

**DOI:** 10.3389/fnut.2019.00186

**Published:** 2019-12-17

**Authors:** Xiaohui Feng, Haili Liu, Zhaoping Li, Arianna Carughi, Sheng Ge

**Affiliations:** ^1^Department of Clinical Nutrition, Shanghai Jiaotong University Affiliated Sixth People's Hospital, Shanghai, China; ^2^Center for Human Nutrition, David Geffen School of Medicine at UCLA, Los Angeles, CA, United States; ^3^American Pistachio Grower, Fresno, CA, United States

**Keywords:** gestational impaired glucose tolerance, gestational diabetes mellitus, pistachio, blood Glucose, insulin, GLP-1, GIP

## Abstract

Long-term consumption of pistachios could potentially improves glucose homeostasis. Impaired postprandial glucose, insulin, and glucagon-like peptide-1 (GLP-1) responses have been reported in gestational diabetes mellitus (GDM) patients. The objective of this study was to evaluate the acute effects of two isocaloric test meals, 42 g pistachios and 100 g whole-wheat bread (WWB) on postprandial glucose, insulin, and gut derived incretin levels in Chinese women with gestational impaired glucose tolerance (GIGT) or GDM. Expected glucose and insulin responses were observed after WWB consumption. Isocaloric pistachio intake had minimal effect on blood glucose or insulin. In both GIGT and GDM patients, significant higher GLP-1 levels were observed at 90 and 120 min after pistachio compared to WWB intake. Significant lower gastric inhibitory polypeptide (GIP) levels were observed at 30 and 60 min in GDM patients or 120 min in GIGT patients after pistachio compared to WWB intake. In summary, isocaloric pistachio intake induced significantly lower postprandial glucose, insulin and GIP but higher GLP-1 levels compared to WWB. Our data suggest pistachios are effective alternative to a low-fat, high-carbohydrate food to improve postprandial glucose, insulin, and GLP-1 response in women with GDM and GIGT.

## Introduction

Gestational diabetes mellitus (GDM) is hyperglycemia with first onset or recognition during pregnancy (1). The prevalence of GDM has increased worldwide in the past decades (2). According to the latest diagnostic criteria established by the International Association of Diabetes and Pregnancy Study Groups (IADPSG) in 2010 (3, 4), the estimated GDM prevalence was at 9.8–25.5% worldwide and 9.3–18.9% in China (5). GDM is known to have a significant impact on the health of both the mother and the baby.

Medical nutrition therapy has been proved to be effective in GDM management (6). The choice of healthy food is the key component. Tryggvadottir et al., showed that adhering to a healthy dietary pattern focusing on seafood, fruits and vegetables, was associated with reduced risk for GDM and GDM associated complications compared to westernized diets, especially among overweight or obese women (7).

Previous epidemiological studies and clinical trials have suggested the metabolic benefits of nut consumption (8). Compared to other nuts, pistachios have a balanced nutrition profile with lower fat [polyunsaturated fatty acids (PUFAs) and monounsaturated fatty acids (MUFAs)], higher protein, fiber (both soluble and insoluble), potassium, phytosterols, γ-tocopherol, vitamin K, and xanthophyll carotenoids (9). Pistachios are also known for their high antioxidant potential (10). Recently, the acute effects of adding pistachios to high carbohydrate foods has been reported and attenuation of postprandial glucose levels in healthy subjects and subjects with metabolic diseases was observed (11, 12). In addition, chronic consumption of pistachios lowered blood glucose levels, LDL-c, and some inflammatory markers in healthy subjects and subjects with metabolic syndrome (13–15).

However, no studies have evaluated the effect of pistachio intake on glucose metabolism in women with GDM. We therefore conducted the first randomized, controlled, crossover study in Chinese women with GDM or GIGT to assess the acute effect of pistachio intake on postprandial glucose, insulin, and gut derived incretin hormones (GLP-1, GIP) in comparison to isocaloric high carbohydrate test meal (whole wheat bread).

## Materials and Methods

### Study Design and Procedures

**Figure 1:**
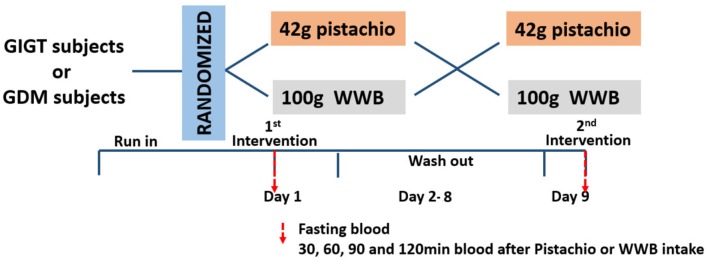
Study design.

This was a randomized, controlled, crossover study comparing the acute response of glucose, insulin and GLP-1 and GIP after consumption of either isocaloric pistachios or high carbohydrate test meal (whole wheat bread, WWB) in women with GIGT or GDM. The protocol was reviewed and approved by the Ethics Committee of Shanghai Sixth People's Hospital. All subjects were given written informed consent in accordance with the Declaration of Helsinki. The study was registered at the Chinese Clinical Trial Registry (http://www.chictr.org.cn, ChiCTR-IOR-17011838). Each intervention was followed by 1-week washout and cross over to the other intervention. Subjects were instructed to consume their usual diets before either of their test days. All subjects were instructed to fast at least 12 h overnight before the test and came in the morning on study days. On the study day, fasting blood samples were collected from subjects to determine baseline levels of glucose, insulin, and incretins. After the collection of fasting blood, participants were provided with either WWB or pistachios and consumed it within 15 min. Blood samples were collected through the indwelling catheter at 30, 60, 90, and 120 min after ingestion of WWB or pistachios (Figure 1).

### Study Participants

Chinese women with an age of 23–39 years old, Han race, singleton pregnancies, and having a gestational age of 24–30 weeks at the time of screening were recruited from the prenatal care clinic in the Shanghai Jiaotong University Affiliated Sixth People's Hospital (Shanghai, China) between June 2016 and March 2017. All subjects underwent universal screening for GDM using a standard 50 g glucose tolerance test at 24–30 weeks' gestation. Plasma glucose concentrations were measured 1 h after giving 50 g glucose and a value of ≥7.8 mmol/L was considered as a positive screening according to recommendations of the 2013 guidelines of gestational diabetes mellitus published by ACOG (American College of Obstetricians and Gynecologists). Women with a positive screening were then proceeded with a 2 h 75 g oral glucose tolerance test (OGTT) after an 8–12 h overnight fasting and the results were interpreted according to the Ministry of Health of the People's Republic of China, 2011 (16). Women fulfilled at least one of the following criteria were diagnosed GDM when fasting glucose ≥5.1 mmol/L, 1 h glucose ≥10 mmol/L or 2 h glucose ≥8.5 mmol/L during the 2 h 75 g OGTT. The diagnosis of GIGT was made when subjects were tested positive with 50-g glucose tolerance test but did not meet the criteria of GDM.

A total of 73 women (34 with the diagnosis of GDM, 39 with GIGT) were recruited, of whom 13 did not meet the inclusion criteria and 1 withdrew from the study. Thirty women with GIGT and 29 with GDM completed the study. Data from 4 GDM participants were excluded due to blood hemolysis.

### Test Meals

Each test meals provided about 240 kcal of energy: 100 g of whole wheat bread (2 slices) (Mankattan, Shanghai, China) or 42 grams of pistachios (Maple, packaged, and distributed by Zhuhai, China, produced in California, USA). The specific nutrients of test meals are listed in Table 1.

**Table 1:** The nutrients of whole wheat bread and pistachios.

**Test food**	**Energy (kcal)**	**Protein (g)**	**Fat (g)**	**CHO (g)**	**Fiber (g)**
WWB (100 g)	249	8.3	3.4	41.9	5.0
Pistachios (42 g)	236	8.8	18.9	12.2	4.2

### Blood Biochemical Analysis

Serum glucose was measured with a type 7600-020 Automated Analyzer (Hitachi, Tokyo, Japan). Serum insulin, GIP, and GLP-1 were measured using the human insulin ELISA kit (Alpco Diagnostics, Salem, NH, USA), and GLP-1 and GIP ELISA Kits (EMD Millipore Corporation, Billerica, MA, USA).

### Statistical Analysis

All the results were analyzed with SPSS 19.0 software package (SPSS Inc. Chicago, IL, USA). Data are expressed as mean ± SEM. Differences in response to test meals (WWB or pistachios) were analyzed by repeated measures ANOVA for main effects of time and test food and the time × test food interaction. If the time × test meal interaction was significant, then paired *t*-tests were conducted for each time point. Results were considered significantly different at *P* < 0.05. The area under the curves (AUC) of blood glucose, insulin, GLP-1 and GIP were analyzed by paired *t*-test or Wilcoxon rank-sum test when data were not normally distributed. Asterisks was used to indicate the significant difference with *P* < 0.05 or *P* < 0.01.

The estimated sample size was *n* = 18. Our sample size calculation was based on Kendall 2014 (12), who compared acute effect of 85 g pistachios to 112 g WWB on GLP-1 response in 20 subjects with metabolic disorders in a crossover design study. We employed the difference of GLP-1 AUC for pistachios and WWB intake to calculate the effect size of 0.9 assuming 0.5 intra class correlation. Therefore, 19 subjects was a sufficient sample size to provide 95% power to detect similar difference. To account for a 10% attrition and complication during pregnancy, we included total of 25 GDM and 30 GIGT patients in this study.

## Results

### Blood Glucose and Insulin

**Figure 2:**
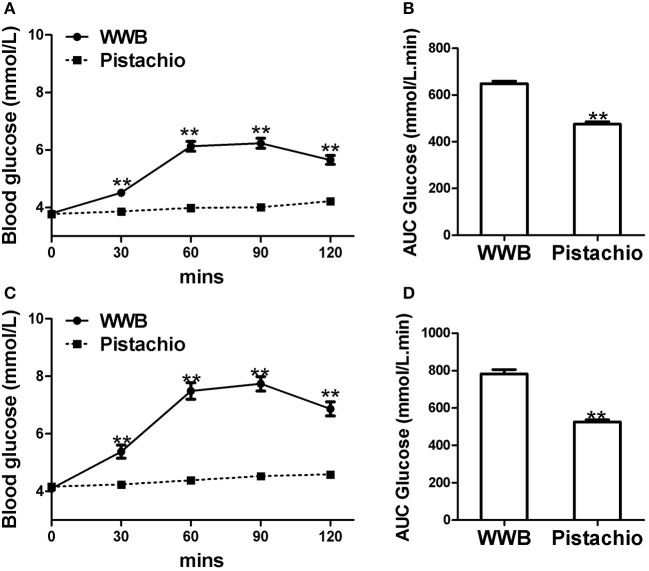
Serum glucose levels and AUC _Glucose0−120min_ in **(A,B)** GIGT participants (*n* = 30) and **(C)** and **(D)** GDM participants (*n* = 25) after a single administration of 100 g whole wheat bread or 42 pistachios. Values are means ± error. Difference was considered significant with ^**^P < 0.01.

**Figure 3:**
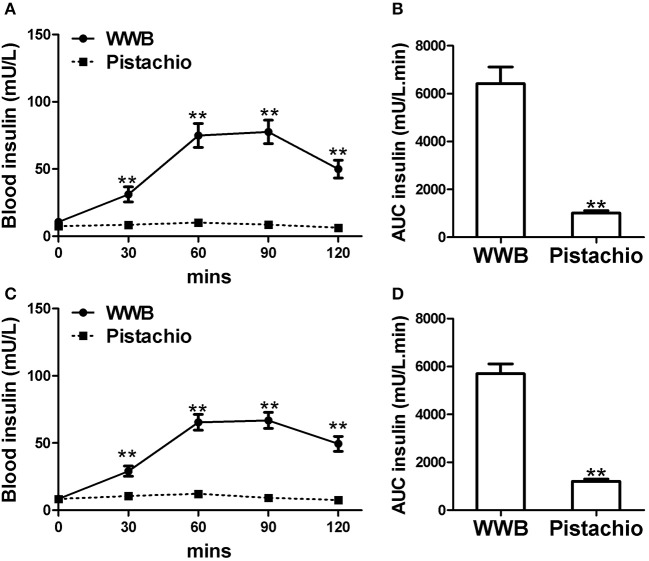
Serum insulin levels and AUC_Insulin0−120min_ in **(A,B)** GIGT participants (*n* = 30) and **(C,D)** GDM participants (*n* = 25) after a single administration of 100 g whole wheat bread or 42 pistachios. Values are means ± error. Difference was considered significant with ^**^P < 0.01.

The demographics of GIGT and GDM participants was shown in Table 2. The response of blood glucose and insulin to two test meals (pistachio vs. WWB) over 2 h period in both GIGT and GDM participants are shown in Figures 2, 3. In both the GIGT and the GDM participants, the baseline blood glucose and insulin levels were similar between two test meals (Figures 2A,C, 3A,C). In GIGT participants, baseline blood glucose levels were at 3.77 ± 0.10 mmol/L for pistachio and 3.79 ± 0.08 mmol/L for WWB. In GDM participants, baseline blood glucose levels were at 4.16 ± 0.10 mmol/L for pistachio and 4.09 ± 0.11 mmol/L for WWB. In GIGT participants, baseline blood insulin levels were at 7.44 ± 0.66 mU/L for pistachio and 10.06 ± 2.14 mU/L for WWB. In GDM participants, baseline blood insulin levels were at 8.34 ± 0.76 mU/L for pistachio and 8.43 ± 0.68 mU/L for WWB.

**Table 2:** Demographics of study participants^a^.

	**GDM (*n* = 25)**	**GIGT (*n* = 30)**
Age (yrs)	31.4 ± 3.0	30.5 ± 2.7
Gestational weeks (wks)	27.0 ± 0.4	26.0 ± 0.4
Height(m)	1.6 ± 0.1	1.6 ± 0.1
BMI (kg/m^2^) (Pre-pregnancy)	21.8 ± 3.2	21.3 ± 2.8
Weight gain(kg) (Final-baseline)	5.0 ± 1.9	4.5 ± 1.7

a *Data are means ± SDs*.

In both GIGD and GDM participants, pistachio consumption did not increase the levels of blood glucose and insulin compared to baseline, while significant increase of blood glucose and insulin was observed after WWB consumption (Figures 2, 3). Compared to pistachio consumption, blood glucose and insulin levels at 30, 60, 90, and 120 min postprandial as well as AUC_glucose0−120min_ and AUC_insulin0−120min_ were significantly higher after WWB consumption in both GIGT and GDM participants (Figures 2, 3).

### Gut Derived Incretins

**Figure 4:**
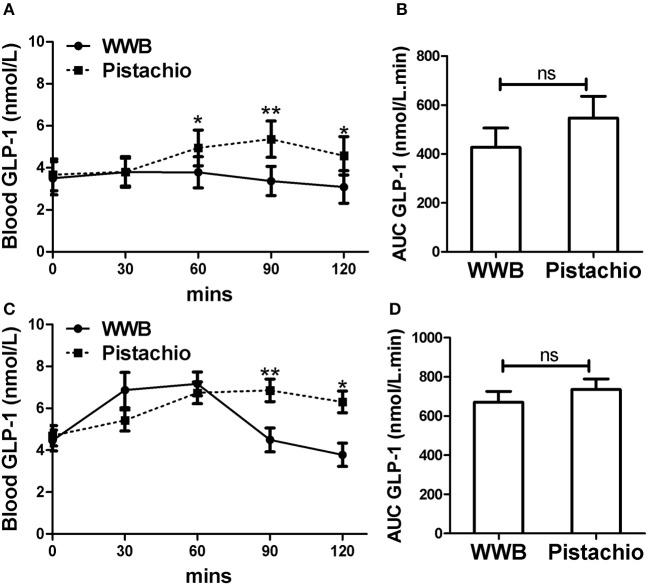
Serum GLP-1 levels and AUC _GLP−10−120min_ in **(A,B)** GIGT participants (*n* = 30) and **(C,D)** GDM participants (n = 25) after a single administration of 100 g whole wheat bread or 42 pistachios. Values are means ± error. Difference was considered significant with ^*^P < 0.05 and ^**^P < 0.01.

**Figure 5:**
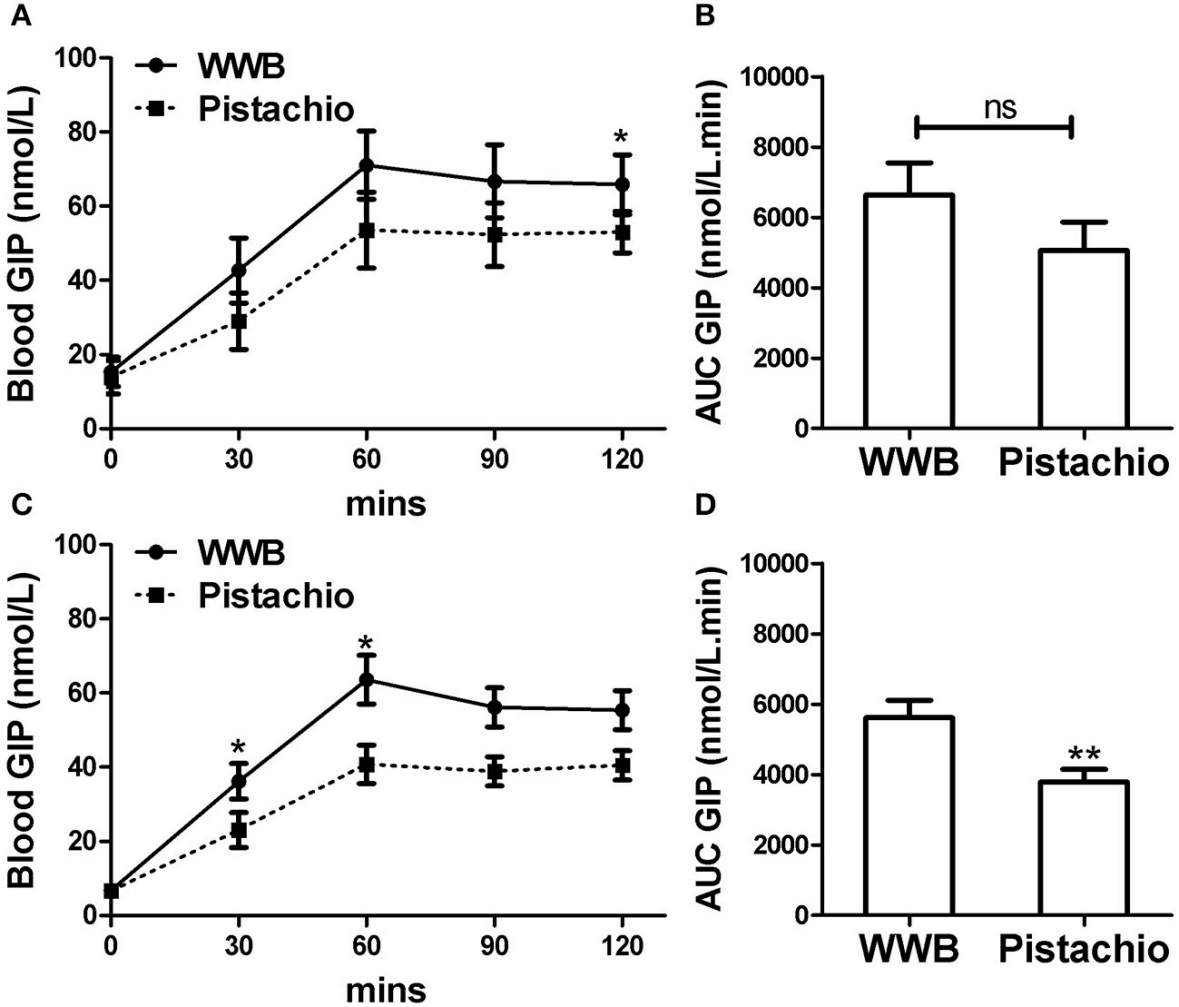
Serum GIP levels and AUC _GIP0−120min_ in **(A,B)** GIGT participants (*n* = 30) and **(C,D)** GDM participants (*n* = 25) after a single administration of 100 g whole wheat bread or 42 pistachios. Values are means ± error. Difference was considered significant with ^*^P < 0.05 and ^**^P < 0.01.

Similar to glucose and insulin, baseline gut derived incretin (GLP-1 and GIP) levels did not differ by test meal assignment (Figures 4, 5). In GIGT participants, baseline blood GLP-1 levels were at 3.67 ± 0.75 pmol/L for pistachio and 3.50 ± 0.79 pmol/L for WWB. In GDM participants, baseline blood GLP-1 levels were at 4.69 ± 0.49 pmol/L for pistachio and 4.46 ± 0.50 pmol/L for WWB. In GIGT participants, baseline blood GIP levels were at 13.91 ± 4.50 pmol/L for pistachio and 15.33 ± 3.98 pmol/L for WWB. In GDM participants, baseline blood GIP levels were at 6.63 ± 0.74 pmol/L for pistachio and 6.75 ± 0.78 pmol/L for WWB.

In GIGT participants, test meal related difference of GLP-1 was observed at 60, 90, and 120 min postprandial, where GLP-1 was increased after pistachio but not WWB consumption (Figure 4A). In GDM participants, test meal related difference of GLP-1 was observed at 90 and 120 min postprandial, where GLP-1 was increased after pistachio but decreased after WWB consumption (Figure 4C).

In both GIGT and GDM participants, blood GIP increased in response to both test meals (Figures 5A,C). The test meal-related difference of GIP was observed at 30 and 60 min postprandial in GDM participants and 120 min postprandial in GIGT participants, where GIP increase was significantly higher after WWB compared to pistachio consumption (Figure 5C).

## Discussion

This is the first study evaluating the acute effect of pistachios on postprandial glucose, insulin, GLP-1 and GIP in women with GDM or GIGT. Currently, WWB and nuts are among the recommended snacks in the 2016 dietary guidelines for pregnant women in China (17). Our results clearly demonstrate that pistachio is a much more favorable snack than WWB with better glycemic control associated with lower insulin levels and improved GLP-1 response.

In both healthy subjects and subjects with metabolic syndromes, significantly improved postprandial glycemia has been reported when white bread was consumed with pistachios compared to white bread alone (11, 12). Hernández-Alonso et al. has found that long-term incorporation of pistachios into regular diets improved the glycemic control and other metabolic markers in prediabetic patients (18). Previous study showed that acute pistachio intake alone had minimal effects on blood glucose and insulin levels in subjects of metabolic syndromes (12). In consistent with previous findings, our data showed that the serum glucose and insulin levels in both GIGT and GDM were not changed within 2 h after pistachio intake while isocaloric WWB led to significant increase of blood glucose and insulin levels. Previous clinical study also reported that increase of fat intake with controlled carbohydrate amount showed a dose dependent reduction of blood glucose with same insulin doses (19). Kendall et al. also showed that better postprandial glucose responses in subjects of metabolic syndromes after pistachio intake compared to white bread intake, with equal carbohydrate content (12). The low carbohydrate, and high fat content of the pistachios likely contributed to the observed differences of postprandial glucose and insulin responses between pistachio and WWB intake. In addition, pistachios are high in carotenoids, which may also contribute to the observed superior postprandial glucose and insulin responses compared to WWB intake. Previous study reported the negative association between levels of plasma carotenoids and the risk for the development of impaired glucose homeostasis and T2DM (20). Other phytonutrients in pistachios, such as ellagitannins, can also possibly affect gastrointestinal sugar absorption and therefore have an impact on postprandial blood glucose levels (21).

The gut incretin hormones GLP-1 and GIP are released to lower blood glucose levels after meal consumption. GLP-1 and GIP also possess strong glucose-dependent insulin regulatory properties and augment glucose-dependent insulin secretion after meal consumption (22). This potentiation of insulin secretion by gut hormones such as GLP-1 after oral glucose load is crucial for controlling postprandial glucose excursions. Impaired postprandial GLP-1 response was previously reported in pregnant women with GDM (23). In subjects with metabolic syndromes, increased levels of GLP-1 and GIP were also observed when white bread was consumed with pistachios compared to white bread alone as well as pistachio alone vs. white bread with equal amount of carbohydrate, suggesting the potential insulin-sparing properties of pistachios (12). In consistent with previous finding, we found that GLP-1 levels, after consuming pistachios, were significantly higher than after consuming WWB both in the GIGT and the GDM groups. The stimulation of GLP-1 release by pistachio intake may be potentially due to the fat load (24). Postprandial GIP levels, however, in our study were lower after consuming pistachios compared to WWB, in both GIGT and GDM subjects. GIP is known for its adipogenic properties in addition to its incretin function, and has been implicated in the connection between high fat diets and the risk of metabolic abnormalities including insulin resistance and type 2 diabetes (25–27). Increased postprandial GIP responses have been reported in patients with type 2 diabetes and recent findings implicate GIP as a “diabetogenic” hormone (25, 28). Therefore, further investigation of long-term sustainability of GLP-1 and GIP stimulation effects of pistachios in GIGT or GDM patients is required to determine whether the acute effects could be translated to long-term improvements.

Chronic inflammation is associated metabolic disorders, including obesity, insulin resistance and diabetes (29). The long-term benefit of diets high in unsaturated fat and fruit/vegetable on the inflammatory state in subjects with metabolic disorders have been widely reported (30). Recent study showed the pomegranate consumption acutely reduced blood proinflammatory cytokine MCP1 levels in healthy subjects (31). Therefore, future study is needed to evaluate the acute effect of pistachio intake on inflammation as well as other metabolic risk markers.

According to the 2013 ACOG guidelines, dividing daily energy intake into three meals and two to three snacks are recommended for women with GDM for the purpose of reducing postprandial glucose fluctuations. The WWB is recommended for pregnant women as a healthy food by Chinese government dietary guidelines as whole grains can help reduce or retard blood glucose responses. We have demonstrated that pistachios had an even lower glycemic response with attenuated insulin response and improved GLP-1 response in both the GIGT group and the GDM subjects. Considering the balanced nutrients, pistachio can be used as a choice for healthy snacks during pregnancy. Further investigation in randomized control trial on long-term consumption of pistachios as well as its the interaction with overall diets is needed.

## Data Availability Statement

All datasets generated for this study are included in the article.

## Ethics Statement

The studies involving human participants were reviewed and approved by Shanghai Jiaotong University Affiliated Sixth People's Hospital. The patients/participants provided their written informed consent to participate in this study.

## Author Contributions

XF and HL performed the study. AC, ZL, and SG designed the project and prepared the manuscript.

### Conflict of Interest

AC is a consultant of American Pistachio Grower. The remaining authors declare that the research was conducted in the absence of any commercial or financial relationships that could be construed as a potential conflict of interest.

## Abbreviations

DM: Gestational diabetes mellitus
GIGT: gestational impaired glucose tolerance
GLP-1: Glucagon-like peptide-1
GIP: Gastric inhibitory polypeptide
WWB: whole wheat bread
IADPSG: International Association of Diabetes and Pregnancy Study Groups
PUFAs: polyunsaturated fatty acids
MUFAs: monounsaturated fatty acids
ACOG: American College of Obstetricians and Gynecologists
AUC: area under the curves.
